# Study on Influence Factors of H_2_O_2_ Generation Efficiency on Both Cathode and Anode in a Diaphragm-Free Bath

**DOI:** 10.3390/ma17081748

**Published:** 2024-04-11

**Authors:** Tian Tian, Zhaohui Wang, Kun Li, Honglei Jin, Yang Tang, Yanzhi Sun, Pingyu Wan, Yongmei Chen

**Affiliations:** College of Chemistry, Beijing University of Chemical Technology, Beijing 100029, China; 2021200907@buct.edu.cn (T.T.); 2020410032@buct.edu.cn (Z.W.); 2023200955@buct.edu.cn (K.L.); 2021210592@buct.edu.cn (H.J.); tangyang@mail.buct.edu.cn (Y.T.); sunyz@mail.buct.edu.cn (Y.S.); pywan@mail.buct.edu.cn (P.W.)

**Keywords:** hydrogen peroxide, carbonate-bicarbonate solution, 2e-ORR pathway, 2e-WOR pathway, coupled electrolytic system

## Abstract

Electrosynthesis of H_2_O_2_ via both pathways of anodic two-electron water oxidation reaction (2e-WOR) and cathodic two-electron oxygen reduction reaction (2e-ORR) in a diaphragm-free bath can not only improve the generation rate and Faraday efficiency (FE), but also simplify the structure of the electrolysis bath and reduce the energy consumption. The factors that may affect the efficiency of H_2_O_2_ generation in coupled electrolytic systems have been systematically investigated. A piece of fluorine-doped tin oxide (FTO) electrode was used as the anode, and in this study, its catalytic performance for 2e-WOR in Na_2_CO_3_/NaHCO_3_ and NaOH solutions was compared. Based on kinetic views, the generation rate of H_2_O_2_ via 2e-WOR, the self-decomposition, and the oxidative decomposition rate of the generated H_2_O_2_ during electrolysis in carbonate electrolytes were investigated. Furthermore, by choosing polyethylene oxide-modified carbon nanotubes (PEO-CNTs) as the catalyst for 2e-ORR and using its loaded electrode as the cathode, the coupled electrolytic systems for H_2_O_2_ generation were set up in a diaphragm bath and in a diaphragm-free bath. It was found that the generated H_2_O_2_ in the electrolyte diffuses and causes oxidative decomposition on the anode, which is the main influent factor on the accumulated concentration in H_2_O_2_ in a diaphragm-free bath.

## 1. Introduction

Hydrogen peroxide possesses a moderate oxidizing capacity with no secondary pollution [1,2], and it is used in various industrial and domestic applications, such as textile and pulp bleaching, waste water treatment, chemical synthesis, circuit board cleaning, clinical disinfection, etc. [3,4,5,6]. The state-of-the-art anthraquinone process for H_2_O_2_ production requires massive infrastructure investment and refined production management, by which an aqueous solution of H_2_O_2_ up to 75 wt% can be obtained while a H_2_O_2_ solution of 30 wt% is marketed and used because of transportation and storage safety [7,8]. However, a low concentration (~3 wt%) is enough in many cases, such as in hospitals and the cosmetics industry, for disinfection and sterilization [9]. For these application situations, preparing H_2_O_2_ solution on-site with a small electrochemical device becomes the most environmentally friendly and economical pathway [10,11,12,13].

There are two electrode reactions of electrosynthesis H_2_O_2_: (1) the two-electron oxygen reduction reaction (2e-ORR) on the cathode with *E*^θ^ = 0.78 V vs. NHE [14,15], and (2) the two-electron water oxidation reaction (2e-WOR) on the anode with *E*^θ^ = 1.76 V vs. NHE [16]. Many 2e-ORR catalytic electrolysis systems with good selectivity, high activity, and good durability have been proposed [17,18,19], but the 2e-WOR catalytic electrolysis system for H_2_O_2_ generation is still unsatisfied in terms of generation rate and Faraday efficiency (FE) [20]. Many efforts have been made to design excellent catalysts to improve the 2e-WOR selectivity [21], to find a suitable electrode substrate, to fasten the catalyst onto the surface of the substrate, and so on [22].

Usually, the counter-electrode reaction for 2e-WOR is the hydrogen evolution reaction (HER), and it is the oxygen evolution reaction (OER) for 2e-ORR [23,24]. Theoretically, if the anodic 2e-WOR and the cathodic 2e-ORR are coupled in one electrolysis bath, the overall FE of H_2_O_2_ generation would be 200%. However, there are several challenges to setting up these coupled electrolytic systems. The primary problem is the distinct electrolyte requirements of the anodic and cathodic catalysts, stemming from the inherently different nature of the two electrode processes. For instance, most metal-based and carbon-based electrocatalysts for 2e-ORR adapt to caustic alkaline electrolytes (i.e., NaOH or KOH solutions) [25,26], but only a few carbon-based electrocatalysts are suitable for weakly alkaline or neutral electrolytes (i.e., Na_2_SO_4_ or PBS solutions) [27,28,29]. In contrast, most of the reported 2e-WOR electrocatalysts with excellent performance require a carbonate electrolyte (Na_2_CO_3_ or NaHCO_3_ solutions) [30,31,32]. Therefore, some studies have setup diaphragm electrolytic systems in which the 2e-ORR and 2e-WOR electrode processes are separated, and the preferential conditions of electrode substrates, catalysts, and supporting electrolytes for each process could be met in this way to achieve optimal productivity of the entire system [33,34].

In contrast to a diaphragm electrolytic system, a diaphragm-free electrolytic system is beneficial for its simple structure, easy operation, omitting electrolyte purification, and eliminating additional diaphragm resistance. However, till now, there have been a few research reports on the H_2_O_2_ generation in diaphragm-free-coupled electrolytic systems [35,36]. The challenges mainly arise from two aspects: (1) using a uniform electrolyte in a bath, which implies that the productivity on either the cathode or the anode would be sacrificed, and (2) the loss of the generated H_2_O_2_ in the electrolyte due to the more concentrated H_2_O_2_ in the cathodic chamber diffusing and going through oxidative decomposition on the anode.

Here we report the study on influent factors of H_2_O_2_ generation efficiency in coupled electrolytic systems. A piece of FTO electrode was used as the anode in this study. And its catalytic performance for 2e-WOR in different electrolytes (i.e., NaOH, Na_2_CO_3_, and mixed Na_2_CO_3_/NaHCO_3_ solutions) was compared. Furthermore, the FTO electrode was coupled with the PEO-CNT cathode. Then, the influent factors on the accumulated concentration of H_2_O_2_ in the electrolyte by a coupled electrolytic system in a diaphragm bath or in a diaphragm-free bath were comparably investigated.

## 2. Materials and Methods

### 2.1. Preparation of Materials and Electrodes

Fluorine-doped tin oxide (FTO) electrode: A piece of FTO-coated glass measuring 20 cm × 20 cm was purchased from MSE Supplies and cut into small pieces measuring 2 cm × 1 cm. It is cleaned by sonication with acetone, ethanol, and water for 15 min each to remove surface impurities. After drying, it is partially covered with 704 silicone rubber to control the working area to 1 cm × 1 cm. Before each electrochemical measurement or electrolysis, the FTO electrode is pre-stabilized at 3.0 V for 20 min in an H-type cell to clean and activate the surface.

Polyethylene oxide-modified carbon nanotubes (PEO-CNTs) loaded electrode: PEO-CNTs were prepared according to the previous report [27] and are briefly described below. The mixture of carbon nanotubes and polyethylene oxide was calcined at 600 °C in an Ar atmosphere for 3 h to get PEO-CNTs. PEO-CNTs was suspended in a mixture of isopropanol and H_2_O (7:3, volumetric ratio), and with the help of some glues (5% Nafion), it was sprayed evenly onto the surface of carbon paper (TORAY, TGP-H-060) with a loading density of 0.5 mg cm^−2^. After drying, it was used as a cathode in the 2e-ORR system.

A piece of hydrophobic carbon fiber paper (CFP) was prepared according to a previous report [36], and it was used as the anode in a flow electrolysis system.

### 2.2. Electrochemical Measurements and Electrolysis Systems

The electrochemical measurements and electrolysis experiments (except for the flow electrolysis system in which a two-electrode system was used) were performed on an electrochemical workstation (CHI 760E, CH Instruments, Chen Hua, Shanghai, China) in a three-electrode system, in which a saturated calomel electrode (SCE) connected by a salt bridge was used as the reference electrode. All potentials described in the text have been converted to the RHE scale using the following Equation (1).

(1) $$E_{\mathrm{RHE}}=E_{\mathrm{SCE}}+0.2438+0.0592\times \mathrm{pH}$$

1. Evaluation of catalytic performance for 2e-ORR

PEO-CNTs suspended in a mixture of isopropanol and H_2_O (7:3 in volume) and adding glues (5% Nafion) were mixed via sonication to get a homogeneous ink. Then the ink was dropped onto the disk electrode (0.2475 cm^2^) of the rotating ring-disk electrode (RRDE) with a loading density of 0.06 mg cm^−2^. The LSV curves were collected in O_2_-saturated solution with a scan rate of 5 mV s^−1^ at 1600 rpm with a platinum sheet as the counter electrode. The applied potential on the Pt ring (0.1866 cm^2^) during the LSV test was fixed at 1.2 V to record the ring current (*I*_ring_). The selectivity for H_2_O_2_ generation (H_2_O_2_ selectivity) in the cathodic ORR process was calculated based on Equation (2).

(2) $$H_{2}O_{2}\;\mathrm{Selectivity}\;(\%)=200\times \frac{I_{\mathrm{ring}}/N_{c}}{I_{\mathrm{disk}}+I_{\mathrm{ring}}/N_{c}}$$

where *I*_ring_ is the ring current, *I*_disk_ is the disk current, and *N*_c_ represents the collection efficiency. The theoretical value of *N*_c_ is 0.37, which is determined by the external dimensions of the ring-disk electrode. In practical application, the value of *N*_c_ was obtained in K_3_[Fe(CN)_6_] after each polishing of the ring-disk electrode.

2. Evaluation of catalytic performance for 2e-WOR

The catalytic performance of FTO electrode in different electrolytes was measured in a H-type glass cell separated by Nafion 117 diaphragm with 8 mL electrolyte in each chamber. The electrolysis was executed in a three-electrode system (WE: FTO 1 cm × 1 cm; CE: platinum sheet, 1 cm × 1 cm × 0.1 cm; RE: SCE) under constant-potential model. The concentration of H_2_O_2_ in electrolyte after electrolysis was determined by KMnO_4_ titration method or Ce^4+^ ultraviolet-visible (UV-vis) spectrophotometric method (see Section 2.3 in detail), and FE for H_2_O_2_ generation is calculated based on Equation (3).

(3) $$\mathrm{FE}(\%)=\frac{2\times c\times V\times F}{Q}\times 100\%$$

where *c* is the determined H_2_O_2_ concentration, *V* is the total volume of the electrolyte, *F* is the Faraday constant and takes the value of 96,485 C mol^−1^, and *Q* is the total amount of passed charge during electrolysis, obtained by integrating the current against time in constant-potential electrolysis.

3. Evaluation of the performance of H_2_O_2_ generation in coupled electrolytic systems

The coupled electrolytic system in a diaphragm bath was setup in the same diaphragm bath for evaluating the performance of 2e-WOR, except that the counter electrode was replaced with a PEO-CNTs loaded electrode (1 cm × 1 cm). After a certain amount of electrolysis, the electrolytes in the cathodic and anodic chambers were respectively sampled to determine the FE of H_2_O_2_ generation on cathode or anode.

The coupled electrolytic system in a diaphragm-free bath was setup in the same H-type glass cell, but no diaphragm was installed, to which 16.00 mL of electrolyte was added. After a certain time of electrolysis under constant potential, the electrolyte was sampled to determine the FE for H_2_O_2_ generation.

The coupled electrolytic system in a diaphragm-free flow electrolytic system was setup in a home-made electrolysis cell with electrolyte flow in and out, with a CFP electrode (the working area is 1 cm × 1 cm) as the anode and PEO-CNTs electrode (the working area is 1 cm × 1 cm) as the cathode. Constant current electrolysis was operated in this two-electrode system with a current density of 80 mA cm^−2^.

### 2.3. Determination of H_2_O_2_ in Electrolytes

The KMnO_4_ titration method or the Ce^4+^ UV-vis spectrophotometric method is selected to determine the H_2_O_2_ concentration, depending on the concentration range. The KMnO_4_ titration method is chosen when the H_2_O_2_ concentration is higher than 50 mM. At this point, this method will use a large volume of KMnO_4_ (>10 mL), which reduces the error due to the estimated reading volume. And if the H_2_O_2_ concentration is in the range of 0.2 mM to 50 mM, the Ce^4+^ UV-vis spectrophotometric method is preferred to provide more accuracy. At this point, the absorbance after the reaction can be between 0.1 and 0.9, and UV spectrophotometer can accurately determine its absorbance. The principle of Ce^4+^ UV-vis spectrophotometric method is as follows: the absorption of Ce^4+^ in a Ce(SO_4_)_2_ standard solution at 320 nm might weaken in the presence of H_2_O_2_ due to the reduction of Ce^4+^ to Ce^3+^, and the change in absorption intensity is in direct proportion to the concentration of H_2_O_2_ [37].

### 2.4. The Analysed Steps of Oxidative Decomposition Rate Constant of H_2_O_2_ on Anodic Surface

**Figure 1:**
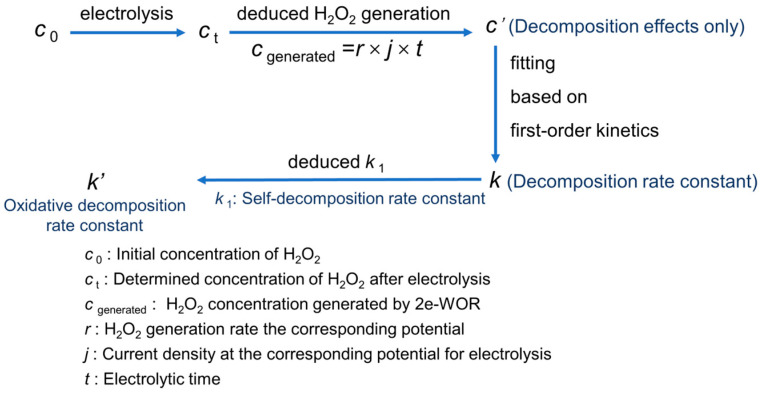
The analysis steps of oxidative decomposition rate constant of H_2_O_2_ at andic surface.

As shown in Figure 1, the expected concentration at a certain moment during the electrolysis would be the sum of the generated H_2_O_2_ via electrode reaction and the added H_2_O_2_ in advance, while the deviation between the actually determined concentration and the expected one is deemed to be the loss of the self-decomposition and the oxidative decomposition. Finally, fitting the relationship between the decreasing concentration and reaction time to a first-order kinetic model, the linear slope (deducted from self-decomposition rate constant *k*_1_) would be the oxidative decomposition rate constant *k*′. Taking the measurement of the oxidative decomposition rate of H_2_O_2_ at 3.6 V in Na_2_CO_3_ solution as an example, the detailed procedure has been described in Supplementary Materials.

## 3. Results and Discussion

### 3.1. FE for H_2_O_2_ Generation via 2e-WOR on FTO in Different Electrolytes

**Figure 2:**
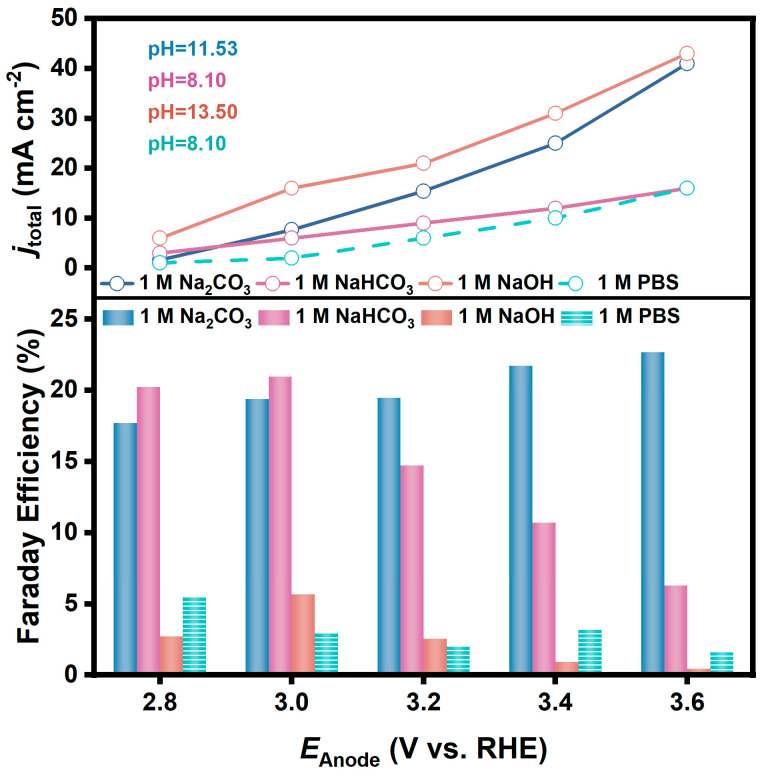
FE and corresponding current densities for H_2_O_2_ generation via 2e-WOR on FTO electrodes in 1 M NaOH (pH = 13.50), 1 M NaHCO_3_ (pH = 8.10), and 1 M Na_2_CO_3_ (pH = 11.53) solutions (vs. PBS solution, pH = 8.10).

With other electrolysis conditions the same, the variations of FE for H_2_O_2_ generation on FTO in 1 M NaOH, 1 M NaHCO_3_, and 1 M Na_2_CO_3_ solutions when electrolyzed at different potentials are shown in Figure 2. It is obvious that the performance in Na_2_CO_3_ solution and NaHCO_3_ solution is significantly better than that in NaOH solution at all potentials. FEs in NaOH were less than 5% at all potentials, but a FE of 21.0% was obtained at 3.0 V in NaHCO_3_, and a FE of 22.7% was obtained at 3.6 V in Na_2_CO_3_.

A PBS solution (pH = 8.10) whose pH is comparable to that of 1 M NaHCO_3_ solution was purposely prepared and used as the electrolyte for the same test, and the FE was only about 5%. It is also noteworthy that the current densities at each potential in these electrolysis tests were in the magnitude of mA cm^−2^. For example, the current density in NaOH solution at 3.0 V reached 16.0 mA cm^−2^, which was much larger than that in Na_2_CO_3_ solution (7.7 mA cm^−2^) and NaHCO_3_ solution (6.0 mA cm^−2^). However, the determined concentrations of H_2_O_2_ in the electrolyte after electrolysis for 20 min were 0.24 mM, 1.02 mM, and 0.95 mM, respectively. It implies that carbonate and bicarbonate solutions are more favorable for H_2_O_2_ generation than alkaline solutions.

**Figure 3:**
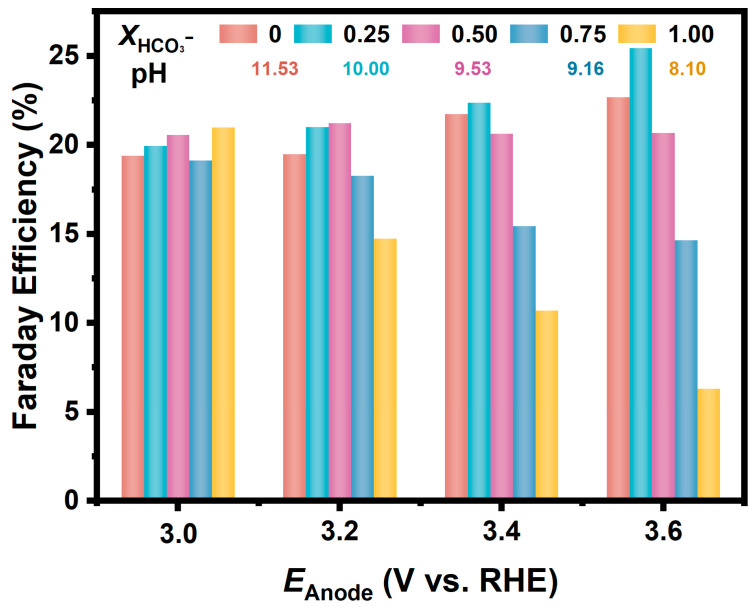
FEs for H_2_O_2_ generation via 2e-WOR on FTO electrodes in CO_3_^2−^/HCO_3_^−^ mixture solutions (pH = 11.53, 10.00, 9.53, 9.16, 8.10) with various ratios at different anodic potentials.

In a further study, we prepared a series of CO_3_^2−^/HCO_3_^−^ solutions with different ratios but held the total concentration of CO_3_^2−^ and HCO_3_^−^ to be 1.0 M. As shown in Figure 3, the Fes for H_2_O_2_ generation on the FTO anode in these solutions are similarly around 20% at relatively low anodic potential (3.0 V). Moreover, in the electrolyte with the molar fraction of HCO_3_^−^ at 0.25, the FE increased as the potential positively shifted, and the FE reached 25.4% at the anodic potential of 3.6 V. While in the electrolyte with a higher proportion of HCO_3_^−^, the FE decreased significantly at a more positive potential; for example, the FE in the NaHCO_3_ solution was only 6% at a potential of 3.6 V.

In the above discussion, the FEs for H_2_O_2_ generation via 2e-WOR were calculated based on the accumulated concentration of H_2_O_2_ in the electrolyte. As shown in Figure 2, the difference among the FEs in different CO_3_^2−^/HCO_3_^−^ solutions became more significant as the potential shifted positively, indicating that the oxidative decomposition of the generated H_2_O_2_ on the anode is a non-negligible factor for the apparent FEs.

The following experiment might provide evidence for this proposal. An amount of 12.5 mM of H_2_O_2_ was respectively added into Na_2_CO_3_ and NaHCO_3_ solutions in advance, and then electrolysis was conducted for 12 min at 3.6 V. As shown in Figure S1 (Supplementary Materials), the H_2_O_2_ concentration in Na_2_CO_3_ increased to 13.4 mM, while that in NaHCO_3_ decreased to 11.0 mM.

### 3.2. Factors Affecting the Accumulated Concentration of H_2_O_2_ in Carbonate Electrolytes

The accumulated concentration of H_2_O_2_ in the electrolyte after electrolysis for a certain time is closely related to the generation of H_2_O_2_ via 2e-WOR on the anode and the decomposition of the generated H_2_O_2_ in the electrolyte. Therefore, in the following text, the factors on FE for H_2_O_2_ generation via 2e-WOR are systematically discussed in kinetic terms.

#### 3.2.1. Self-Decomposition Rate of H_2_O_2_ in Different Solutions

Many groups have reported the various kinetic models of H_2_O_2_ decomposition in different solutions [38,39,40,41,42]. At least one point of consensus can be obtained: the self-decomposition reaction of H_2_O_2_ in aqueous solution follows first-order kinetics, but the rate constant *k_1_* is related to the species and concentration of coexisting chemicals in the solution because some chemicals act as catalysts or inhibitors for H_2_O_2_ decomposition.

**Figure 4:**
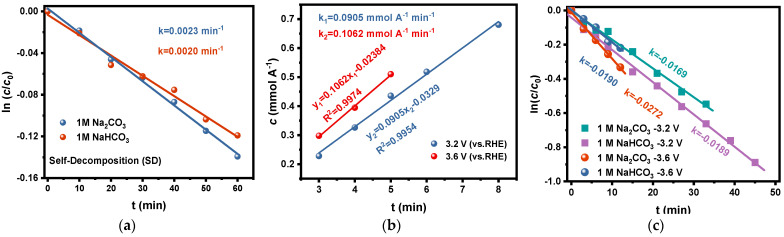
(**a**) The rate constants of H_2_O_2_ self-decomposition in 1 M Na_2_CO_3_ and 1 M NaHCO_3_ solutions at room temperature (20 °C); (**b**) The rate constants of H_2_O_2_ generation via 2e-WOR on FTO electrode at potentials of 3.2 V and 3.6 V in 1 M Na_2_CO_3_ solution; (**c**) The rate constants of H_2_O_2_ oxidative decomposition on FTO at 3.2 V and 3.6 V potentials in 1 M Na_2_CO_3_ and 1 M NaHCO_3_ solutions.

To avoid the interference of the stabilizers in the commercially available H_2_O_2_ reagents on the test results in this study, we used the electrolytes after electrolysis in the carbonate electrolytes as the initial solutions and determined the change in H_2_O_2_ concentration with time at room temperature (20 °C). The curve of ln(*c*/*c*_0_)-*t* is plotted as shown in Figure 4a, which showed that the rate constants *k_1_* of H_2_O_2_ self-decomposition in Na_2_CO_3_ and NaHCO_3_ solutions were 0.0023 min^−1^ and 0.0020 min^−1^, respectively. Based on the above discussion, the effect of self-decomposition on the accumulated concentration of H_2_O_2_ during electrolysis could be ignored because both the rate constant *k_1_* and the H_2_O_2_ concentration in the electrolyte are relatively low, especially at the beginning of the electrolysis.

#### 3.2.2. Generation Rate of H_2_O_2_ via 2e-WOR on Anode

Supposing no decomposition of H_2_O_2_ occurs during the electrolysis, the partial current density for H_2_O_2_ generation (*j*_H2O2_) would be the same at any moment during a constant-potential electrolysis using a given anode in a certain electrolyte. So, the generation rate (*r*) of H_2_O_2_ via 2e-WOR on the anode might be described as Equation (4), which is the differential equation of Equation (3).

(4) $$r=\frac{dc}{dt}=\frac{j_{H_{2}O_{2}}}{V\cdot 2\cdot F}$$

This means that the accumulated concentration (*c*) in the electrolyte would increase linearly with the electrolysis time (*t*), in which the coefficient of *c*-*t* (with the dimension of mmol A^−1^ min^−1^) might be a kinetic indicator for the electrode reaction whose value is dependent on the catalyst, the electrolyte, the electrolytic potential, and so on.

We tried to estimate the generation rate of H_2_O_2_ via 2e-WOR by the following method: The accumulated concentrations of H_2_O_2_ in Na_2_CO_3_ solution after electrolysis at different potentials for a short time (about 3 to 8 min) were determined. In this case, it is reasonable that the decomposition of H_2_O_2_ in the electrolyte can be ignored since the total concentration is low at the beginning of electrolysis. Based on this supposition, the coefficients of *c*-*t* of H_2_O_2_ generation via 2e-WOR on the FTO electrode in Na_2_CO_3_ were estimated to be 0.0905 mmol A^−1^ min^−1^ at 3.2 V and 0.1062 mmol A^−1^ min^−1^ at 3.6 V (Figure 4b), respectively. The results conform to the basic principle of electrochemistry: that the anodic process is faster and has more positive potential.

We also determined the generation rate in various HCO_3_^−^/CO_3_^2−^ solutions and found that the coefficients of *c*-*t* were basically unchanged at the same anodic potential (see the details in Table S1, Supplementary Materials), which can be explained by the fact that the H_2_O_2_ generation rate in the carbonate electrolyte is dependent on the formation rate of the percarbonate ions (HCO_4_^−^) [43], which would be unchanged in carbonate solutions with the same total concentration of CO_3_^2−^ and HCO_3_^−^ because of the quick interconversion equilibrium between CO_3_^2−^ and HCO_3_^−^.

#### 3.2.3. Oxidative Decomposition Rate of H_2_O_2_ on the Anodic Surface

According to basic electrochemistry, it is unavoidable that the generated H_2_O_2_ in the electrolyte diffuses to the anode and goes through oxidative decomposition on it during the electrolysis because the anodic potential is more positive than 3.0 V and the oxidation potential of H_2_O_2_ is about 1.76 V. In this study, the oxidative decomposition rate of H_2_O_2_ at a certain potential is estimated by a deduction method, in which a certain amount of H_2_O_2_ is added to the electrolyte before the electrolysis to magnify the effect of oxidative decomposition. The method is briefly described in Section 2.4.

Based on this method, the rate constants of oxidative decomposition of H_2_O_2_ on the FTO electrode are determined as 0.0146 min^−1^ at 3.2 V and 0.0167 min^−1^ at 3.6 V in Na_2_CO_3_ solution, 0.0169 min^−1^ at 3.2 V, and 0.0252 min^−1^ at 3.6 V in NaHCO_3_ solution, respectively (shown in Figure 4c). It is easy to understand that the oxidative decomposition rate of H_2_O_2_ increases on anodes with more positive potential. What is noticeable is that the oxidative decomposition rate of H_2_O_2_ in NaHCO_3_ is higher than that in Na_2_CO_3_, and this trend became more significant at more positive anodic potentials. This phenomenon is in conformity with the previous studies by Wang et al. [43] and by Zheng et al. [44]. The possible reason is that the acidification near the anode due to OER, especially at a more positive potential, promotes the hydrolysis of HCO_4_^−^ to H_2_O_2_, and the latter is more likely to go through oxidative decomposition than HCO_4_^−^ species [45].

Based on the above discussions, the apparent Fes of H_2_O_2_ generation on the FTO electrode in the carbonate electrolyte shown in Figure 2 can be rationalized. Since the self-decomposition rate of H_2_O_2_ in electrolyte can be ignored, and the generation rate is only related to the anodic potential, the main factor affecting the accumulated concentration of H_2_O_2_ in electrolyte during electrolysis is oxidative decomposition. In carbonate electrolytes with a higher ratio of HCO_3_^−^ and a more positive potential, the oxidative decomposition rate of H_2_O_2_ is higher, which results in a decrease in the apparent Fes.

### 3.3. Efficiency of H_2_O_2_ Generation via 2e-ORR in Carbonate Solutions

PEO-CNTs have been proven to be an effective catalyst for 2e-ORR in a caustic electrolyte by our previous studies, which demonstrated a selectivity better than 85% in a 1 M NaOH solution at a cathodic potential of 0.4 V [27]. In this study, we investigated the changes in the catalytic performance of 2e-ORR in different electrolytes by using PEO-CNTs as cathodic catalysts.

**Figure 5:**
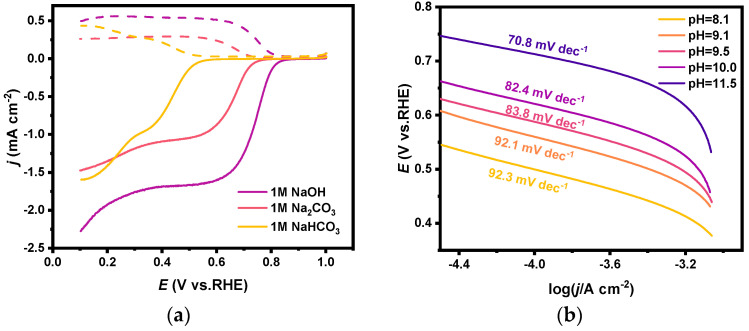
(**a**) RRDE polarization curves of PEO-CNTs loaded electrodes in 1 M NaOH, 1 M Na_2_CO_3_, and 1 M NaHCO_3_ solutions, where the dashed line is the ring current and the solid line is the disk current; (**b**) Tafel curves of PEO-CNTs loaded electrodes in Na_2_CO_3_/NaHCO_3_ mixed solutions and the corresponding slope.

The catalytic performance for 2e-ORR of PEO-CNTs in different solutions (i.e., 1 M NaOH, 1 M Na_2_CO_3_, and 1 M NaHCO_3_) was investigated by the RRDE method. As shown in Figure 5a, the H_2_O_2_ selectivity in all three electrolytes is around 85 ± 5% (the electron transfer number is calculated as 2.3) at potentials in the range of 0.3 V to 0.5 V (Figure S2, Supplementary Materials). However, the onset potentials of the ring current in Na_2_CO_3_ and NaHCO_3_ solutions were significantly negatively shifted (0.55 V) compared with that in NaOH solution, which indicates that the catalytic activity of PEO-CNTs in carbonate electrolyte is weaker than that in caustic electrolyte.

Tafel and other electrochemical methods were conducted to explore the reasons for the decrease in the catalytic activity of 2e-ORR in different electrolytes. The LSV became almost the same after being normalized when different concentrations of equal amounts of Na_2_CO_3_/NaHCO_3_ were added to a 1 M KOH solution [46] (see Figure S3, Supplementary Materials). And it proves that the decrease in 2e-ORR catalytic activity in carbonate electrolytes is partly due to the decreased solubility of oxygen gas in the electrolytes. The measured Tafel slopes in carbonate solutions with different ratios of CO_3_^2−^/HCO_3_^−^ (i.e., with the same concentration of Na_2_CO_3_ and NaHCO_3_ mixed solutions and at different pH) are shown in Figure 5b. The Tafel slope increased from 70.8 mV dec^−1^ to 92.3 mV dec^−1^ with decreasing pH, indicating that the reaction resistance on the electrode surface increased as the pH in the electrolyte decreased. It might be explained that less concentrated OH^−^ ions in the electrolyte hindered the rate of 2e-ORR, of which the protonation process in the first electron transfer step is the rate-controlling step.

### 3.4. The Efficiency of H_2_O_2_ Generation in a Coupled Electrolytic System

**Figure 6:**
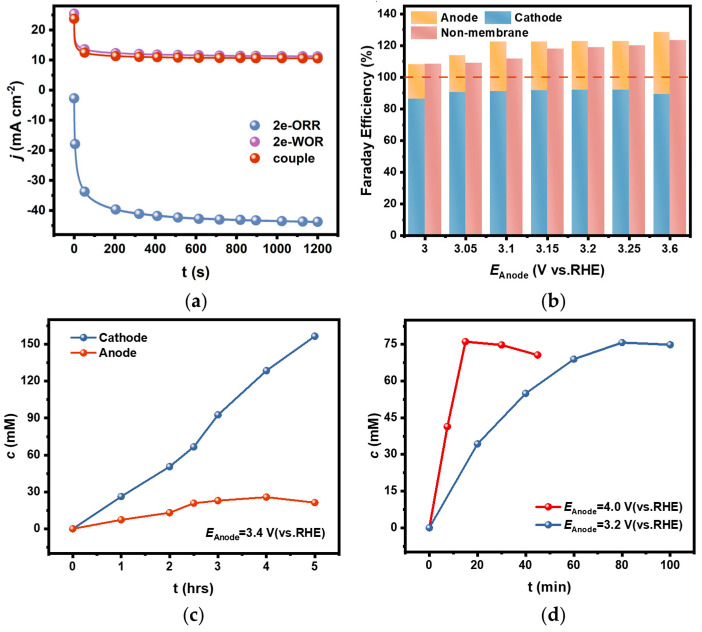
(**a**) The comparison of current densities during electrolysis in 2e-WOR, 2e-ORR, and diaphragm-coupled electrolytic systems under constant potential models; (**b**) The comparison of determined FEs for H_2_O_2_ generation in CO_3_^2−^/HCO_3_^−^ mixed solution in a diaphragm bath and diaphragm-free bath; (**c**) The accumulated concentration of H_2_O_2_ in cathodic and anodic chambers with electrolysis time in the diaphragm-coupled system; (**d**) The accumulated concentration of H_2_O_2_ in the diaphragm-free-coupled system at different potentials.

A coupled electrolytic system for H_2_O_2_ generation in a diaphragm bath was set up by using an FTO electrode as anode, a PEO-CNTs-loaded electrode as cathode, and a 2 M CO_3_^2−^/HCO_3_^−^ solution (mole ratio of CO_3_^2−^:HCO_3_^−^ = 3:1) as the supporting electrolyte. The electrolysis was carried out under the constant potential model. It was found that the actual current density in the bath was only about 10 mA cm^−2^ when the potential was kept constant at 3.0 V (Figure 6a). This current density is comparable to the value when HER occurs on the corresponding cathode at the same anodic potential in a diaphragm bath, while the current density on the PEO-CNTs-loaded cathode could be up to 40 mA cm^−2^ when OER occurs on the corresponding anode. It appeared that the actual current density in a coupled electrolytic system for H_2_O_2_ generation was limited by the sluggish 2e-WOR on the anode, and the catalytic activity for 2e-ORR on the cathode was severely inhibited.

However, it is fortunate that the selectivity for H_2_O_2_ generation both on the cathode and the anode remained in the coupled electrolytic system. The FEs were calculated based on the accumulated H_2_O_2_ concentration in the separated cathode and anode chambers after electrolysis for a short time of 20 min, as shown in Figure 6b. It showed that the FEs were above 85% on the cathode and above 20% on the anode in the diaphragm-coupled electrolytic system, which are comparable to the values in the uncoupled systems. In other words, the total FE of H_2_O_2_ generation on both the cathode and anode in a diaphragm bath could exceed 100%.

The accumulated concentrations of H_2_O_2_ in the cathode and anode chambers were tracked, respectively. The H_2_O_2_ concentration in the cathode chamber increased linearly with electrolytic time and reached 155 mM after electrolysis for 5 h. Whereas, the H_2_O_2_ concentration in the anode chamber increased slower than that in the cathode chamber, and it started to decrease when the concentration reached 26 mM after 3 h of electrolysis (Figure 6c). This phenomenon is related to the low efficiency of H_2_O_2_ generation via 2e-WOR on the anode, and the oxidative decomposition rate accelerated as the concentration increased.

The coupled electrolytic system for H_2_O_2_ generation in a diaphragm-free bath was also setup, in which all the electrolysis conditions were the same as those in the diaphragm bath except for the diaphragm being removed. The FEs determined by the H_2_O_2_ concentration in the electrolyte after a short time of electrolysis are shown in Figure 6b. The total FE in the diaphragm-free bath was slightly lower than that in the diaphragm bath because the H_2_O_2_ concentration was decreased by the anodic oxidative decomposition since there was no diaphragm to avoid the generated H_2_O_2_ via 2e-ORR diffusing to the anode. But it still exceeded 100%, and the cell voltage in the diaphragm-free electrolytic system was lowered compared with the diaphragm-coupled system (see the data in Table S4, Supplementary Materials), which might be the result of removing the diaphragm resistance.

It is worth noting that the anodic oxidative decomposition significantly affected the accumulated concentration in the diaphragm-free electrolytic system for a longer electrolysis time. As shown in Figure 6d, the H_2_O_2_ concentration in the electrolyte decreased as the electrolysis time extended. The inflection point appeared earlier when electrolysis was conducted at an anodic potential of 4.0 V than at 3.2 V, and the maximum concentration was about 75 mM in both cases. This phenomenon can be explained by the fact that the increased generation rate at higher anodic potentials simultaneously accelerates the oxidative decomposition rate as the concentration increases.

The above results show that the oxidative decomposition of the generated H_2_O_2_ in the electrolyte during the electrolysis is the main factor troubling the efficiency of H_2_O_2_ generation by the coupled electrolytic system in a diaphragm-free bath. The following strategies might be taken to solve this problem: One strategy is to find an excellent catalyst for 2e-WOR, i.e., at a lower anode potential and/or with a larger current density, by which to increase the H_2_O_2_ generation rate on the anode and simultaneously decrease the oxidative decomposition rate. The other strategy is to adopt a flow bath, in which the electrolyte could flow out of the bath in time when the accumulated concentration reaches a certain value to avoid anodic oxidative decomposition.

**Figure 7:**
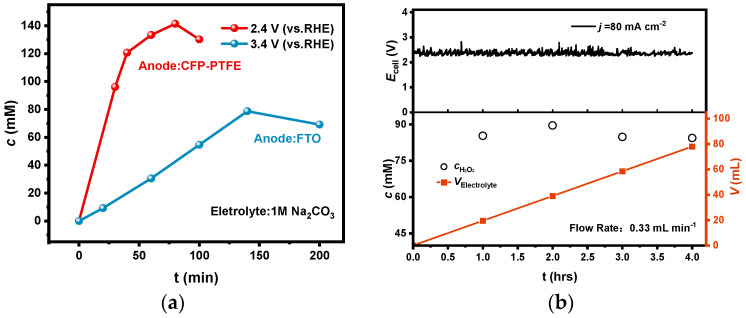
(**a**) The accumulation concentration of H_2_O_2_ in a CFP-PTFE/FTO diaphragm-free system by a long electrolysis time; (**b**) H_2_O_2_ concentration, accumulated volume, and cell potential in the flow bath with a flow rate of 0.33 mL min^−1^ at a constant current of 80 mA cm^−2^.

A CFP electrode has been reported to possess good catalytic performance for 2e-WOR, but its durability is not satisfied. As shown in Figure 7a, using a CFP electrode as anode in a diaphragm-free bath coupled with a PEO-CNTs-loaded electrode, the electrolysis could be operated at an anodic potential of 2.4 V with a large current density of 80 mA cm^−2^, but the performance degraded after electrolysis for only 1.5 h. It is noticeable that the inflection points of the accumulated concentration in electrolyte also appeared; the maximum concentration improved to 146 mM due to the lower anodic potential.

Furthermore, we established a diaphragm-free flow bath with an electrode effective area of 1 cm^2^, and electrolysis was carried out under the constant current model at a current density of 80 mA cm^−2^ and a flow rate of 0.33 mL min^−1^. As shown in Figure 7b, the H_2_O_2_ concentration in the effluent was basically maintained at about 85 mM during electrolysis for 4 h.

## 4. Conclusions

The generation of H_2_O_2_ in a diaphragm-free bath by coupling the 2e-ORR pathway on the cathode and the 2e-WOR pathway on the anode has the advantages of simple device structure and energy savings, while the challenges are the choice of the electrolyte and how to avoid the oxidative decomposition of the generated H_2_O_2_ on the anode. The following results have been obtained in this study:

(1) The effect of the electrolyte on the H_2_O_2_ generation efficiency via 2e-WOR was investigated by using the FTO electrode as an anode in different electrolytes. The highest FE was observed in a mixed Na_2_CO_3_/NaHCO_3_ solution, and the reason is attributed to the oxidative decomposition of generated H_2_O_2_ on the anode being slow in this electrolyte.
(2) The comparison of the coupled electrolytic system in a diaphragm bath and a diaphragm-free bath was performed. The results showed that the oxidative decomposition of generated H_2_O_2_ on the anode was the main reason for the decrease in accumulated concentration in the diaphragm-free electrolytic system.
(3) A diaphragm-free coupled flow electrolysis system was developed to reduce anodic potential and prevent excessive accumulated concentration. This system successfully achieved the continuous generation of an 85 mM H_2_O_2_ concentration at a constant flow rate.

The results of this study might lighten the application of diaphragm-free coupled systems for H_2_O_2_ generation in the future, in which an excellent catalyst for the 2e-WOR pathway and a well-designed diaphragm-free electrolytic system are prerequisites.

## Data Availability

Data are contained within the article and Supplementary Materials.

## Supplementary Materials

The following supporting information can be downloaded at: https://www.mdpi.com/article/10.3390/ma17081748/s1, Figure S1: H_2_O_2_ concentration changed with electrolytic time, adding 12.5 mM H_2_O_2_ as initial solutions; Table S1: H_2_O_2_ concentration by constant-potential (2.8 V) electrolysis with 20 min in 1 M Na_2_CO_3_ solution and a mixed CO_3_^2−^/HCO_3_^−^ (mole ratio of CO_3_^2−^:HCO_3_^−^ = 3:1) solution (the concentration of CO_3_^2−^ and HCO_3_^−^ is 1 M); Table S2: Taking the measurement of the oxidative decomposition rate of H_2_O_2_ at 3.6 V in Na_2_CO_3_ solution; Figure S2: H_2_O_2_ selectivity and electro transfer number of PEO-CNTs in different electrolytes; Figure S3: (a) LSV of PEO-CNTs in 1 M KOH, adding the different mixed solution of Na_2_CO_3_/NaHCO_3_; (b) normalized current of PEO-CNTs in 1 M KOH, adding the different mixed solution of Na_2_CO_3_/NaHCO_3_; Table S3: Physical properties of (A) 1 M KOH, 0.01 M Na_2_CO_3_, 0.01 M NaHCO_3_; (B) 1 M KOH, 0.05 M Na_2_CO_3_, 0.05 M NaHCO_3_; (C) 1 M KOH, 0.1 M Na_2_CO_3_, 0.1 M NaHCO_3_; Table S4: The cell voltage of diaphragm bath systems and diaphragm-free bath systems.
