# Supplementary material for: Study on Influence Factors of H2O2 Generation Efficiency on Both Cathode and Anode in a Diaphragm-Free Bath
Source: Materials (Basel). 2024 Apr 11;17(8):1748. doi: 10.3390/ma17081748 (PMC11050835; doi:10.3390/ma17081748)
Supplement: Supplementary file 1 [file materials-17-01748-s001.zip › materials-2932874-supplementary.pdf]

materials MDPI

Supplementary Materials

Study on Influence Factors of H_2O_2 Preparation Efficiency on Both Cathode and Anode in a Diaphragm-Free Bath

Tian Tian, Zhaohui Wang, Kun Li, Honglei Jin, Yang Tang, Yanzhi Sun, Pingyu Wan and Yonemei Chen *

College of Chemistry, Beijing University of Chemical Technology, Beijing 100029, China

* Correspondence: chenym@mail.buct.edu.cn; Tel.: +10-64435452

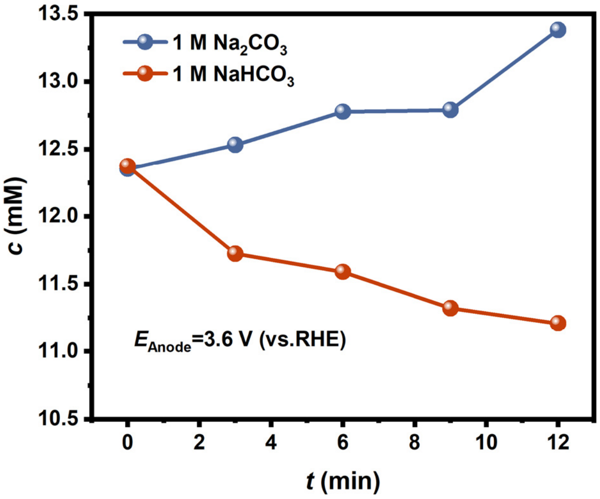

Figure S1. H_2O_2 concentration changed with electrolytic time, adding 12.5 mM H_2O_2 as initial solutions.

Table S1. H_2O_2 concentration by constant-potential (2.8 V) electrolysis with 20 min in 1 M Na_2CO_3 solution and a mixed CO_3^{2-}/HCO_3^- (mole ratio of CO_3^{2-}: HCO_3^- = 3:1) solution (the concentration of CO_3^{2-} and HCO_3^- is 1 M).

Electrolyte	$c_{H_2O_2}$ (mM)
1 M Na_2CO_3	0.267
1 M Na_2CO_3/Na_2HCO_3	0.271

Taking the measurement of the oxidative decomposition rate of H_2O_2 at 3.6 V in Na_2CO_3 solution as an example:

Generation of H_2O_2 after electrolysis with certain time is calculated based on the equation (S1):

$$c_{\text{generated}} = (0.1062 \text{ mmol A}^{-1} \text{ min}^{-1} \times t - 0.0238 \text{ mmol A}^{-1}) \times j \times S_{work} \quad \text{(S1)}$$

where 0.1062 mmol A^{-1} min^{-1} is generation rate of H_2O_2 at 3.6 V in Na_2CO_3 solution, 0.0238 mmol A^{-1} is the parameter, due to the fact that the amount of electricity used to form the double layer capacitor at the beginning of the reaction did not generate H_2O_2, j is current density, t is electrolytic time, and S_{work} is working area (1 cm^2).

The remaining concentrations after electrolysis with certain time (3 min, 6 min, 9min, and 12 min) at 3.6 V were determined based on the initial H_2O_2 concentration of 12.351 mM, which resulted from a combination of generation and decomposition, and the H_2O_2 concentrations with only decomposition factors were calculated based on the equation (S2).

$$c'_{\text{only decomposition}} = c_{\text{determined}} - c_{\text{generated}} \quad \text{(S2)}$$

And then, fitting the relationship between the decreasing concentration and reaction time to a first-order kinetic model ($\frac{d_c}{d_t} = kc$), and we plot and fit a straight line in ln (c'/c_0')-t. The linear slope would be the decomposition rate constant k_d, Finally, the oxidative decomposition rate constant k' was calculated based on the equation (S3).

$$k' = k_d - k_1, \quad \text{(S3)}$$

Table S2. Taking the measurement of the oxidative decomposition rate of H_2O_2 at 3.6 V in Na_2CO_3 solution.

t (min)	$c_{\text{determined}}$	$c_{\text{generated}}$	$c'_{\text{only decomposition}}$	ln (c'/c_0')
0	12.351	0	12.351	0
3	12.530	0.775	11.755	-0.049
6	12.777	1.553	11.224	-0.0957
9	12.789	2.432	10.357	-0.176
12	13.381	3.346	10.035	-0.208

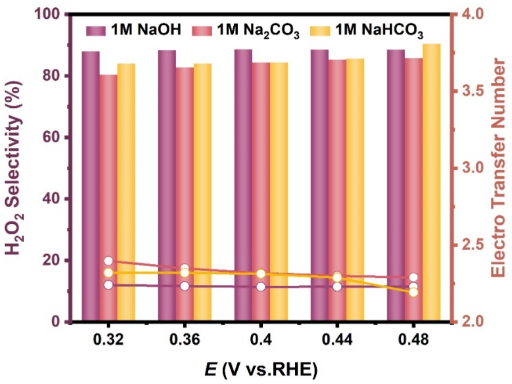

Figure S2. H_2O_2 selectivity and electro transfer number of PEO-CNTs in different electrolytes.

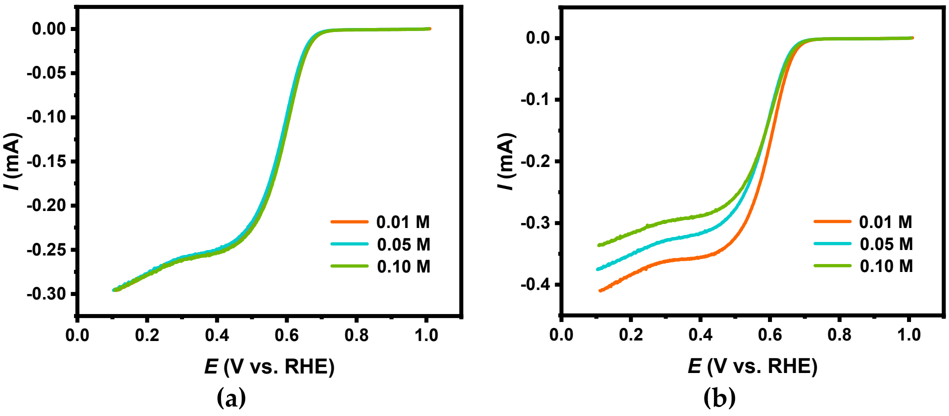

Figure S3. (**a**) LSV of PEO-CNTs in 1 M KOH adding the different mixed solution of Na_2CO_3/$NaHCO_3$; (**b**) Normalized current of PEO-CNTs in 1 M KOH adding the different mixed solution of Na_2CO_3/$NaHCO_3$.

Current normalization

For a rotating disk electrode experiment the observed current is expressed by

$$\frac{1}{i}=\frac{1}{i_K}+\frac{1}{i_L}=\frac{1}{i_K}+\frac{1}{B\omega^{1/2}} \tag{S4}$$

where i is the observed current, i_k is the kinetic current and i_L is the mass transport limited current. Mass transport limited currents for a rotating disk electrode are described by the Levich equation:

$$i_L=0.62nFAD_O^{2/3}\omega^{1/2}v^{-1/6}c_O=B\omega^{1/2} \tag{S5}$$

where n is the overall number of electrons transferred during reaction, F is the Faraday constant, A is the electrode active area, D is the oxygen diffusion coefficient, ω is the rotation rate, v is the kinematic viscosity and C is the dissolved oxygen concentration. B is expressed by

$$B=0.62nFAD_O^{2/3}v^{-1/6}c_O, \tag{S6}$$

Table S3. Taking the measurement of the oxidative decomposition rate of H_2O_2 at 3.6 V in Na_2CO_3 solution.[1].

Electrolyte	pH	v (cSt)	c_{oxygen}	D 10^5 cm^2 s^{-1}
A	13.70	0.93	0.86	2.04
B	13.64	0.96	0.81	1.94
C	13.46	1.0	0.76	1.83

Since oxygen concentration and diffusion coefficient decrease and kinematic viscosity increases when more salt is present in solution (Table S3). To provide a balanced comparison between the RRDE disk plots, it is useful to normalize the observed current to the oxygen diffusion coefficient, electrolyte kinematic viscosity and the oxygen saturation concentration, which can be done by

$$i_{normalized}=\frac{i_{observed}}{D_O^{2/3}v^{-1/6}c_O}=0.62nFA \tag{S7}$$

Table S4. The cell voltage of diaphragm bath system and diaphragm-free bath system.

E (V vs. RHE)	Q (C)	E_{cell} (V)	
3.05	14.311	3.0	
	13.901	2.9	Diaphragm-free
3.15	21.326	3.3	
	20.433	3.2	Diaphragm-free

References

1. Vega, J.A.; Mustain, W.E. Effect of CO_2, HCO_3^- and CO_3^{2-} on Oxygen Reduction in Anion Exchange Membrane Fuel Cells. *Electrochimica Acta* **2010**, *55*, 1638–1644.

Disclaimer/Publisher's Note: The statements, opinions and data contained in all publications are solely those of the individual author(s) and contributor(s) and not of MDPI and/or the editor(s). MDPI and/or the editor(s) disclaim responsibility for any injury to people or property resulting from any ideas, methods, instructions or products referred to in the content.
